# Exploring the scientific underpinnings of investigating safety signals: analytical insights in deciphering drug safety evidence

**DOI:** 10.3389/fdsfr.2024.1445998

**Published:** 2024-08-16

**Authors:** Tarek A. Hammad, Simon Davis, Salman Afsar

**Affiliations:** ^1^ Medical Safety of Marketed Products Development and Plasma-Derived Therapies, Patient Safety and Pharmacovigilance, Takeda Pharmaceuticals Inc., Cambridge, MA, United States; ^2^ Statistical and Quantitative Sciences, Data Sciences Institute, Takeda Pharmaceuticals Inc., Cambridge, MA, United States; ^3^ Medical Safety Assessment Physician and Program, Worldwide Patient Safety, Bristol Myers Squibb, Princeton, NJ, United States

**Keywords:** drug safety, pharmacovigilance, analytical insights, evidence interpretation, safety signals assessment

## Abstract

Ensuring the safety of drugs is a critical aspect of healthcare. Accurate interpretation of drug safety evidence is vital to understand the safety profile and to evaluate the benefits and risks of the medicinal product. However, validity of this evidence has numerous challenges that must be carefully considered, highlighting the need for a heightened appreciation of data interpretation pitfalls. This paper aims to delve into the intricacies of analytic considerations in drug safety data interpretation aiming at providing insights into the safety profile of pharmaceutical interventions. The applicability of these analytic considerations extends to controlled and observational data as well as spontaneously reported individual case reports. Increasing the understanding of scientific underpinnings of evidence interpretation empowers Pharmacovigilance professionals to grasp fundamental concepts, use appropriate terminology, engage in meaningful discussions with colleagues conducting analyses, and critically evaluate evidence. These skills and knowledge enable them to make informed decisions and enhance their expertise in drug safety. By correct utilization of analytic approaches while appreciating their strengths and limitations, one can advance drug safety and benefit-risk research using evidence-based decision-making and ultimately ensure better healthcare outcomes for patients.

## 1 Introduction

Ensuring the safety of drugs is of paramount importance in healthcare. Robust and accurate interpretation of drug safety data plays a major role in assessing the benefit risk profile of the medicinal product. Investigating drug safety findings optimally is not only a mere assessment of causal associations between the products and adverse events, but it also involves characterizing the different aspects of their associations based on the available data. To gain a comprehensive understanding of drug safety, it is important to delve into the nuances of evidence underlying these associations as it holds significant value for patients, healthcare practitioners, and regulators in making informed treatment decisions.

Conceptually, some of the topics discussed in this paper may not be new to the readers. However, this paper adopts a drug safety perspective to explore these topics. Given the scarcity of safety endpoints, the sources of inaccuracy in evidence interpretation might have a larger impact on the benefit-risk conclusion. Since methodological concepts can present challenges for some healthcare professionals who are not accustomed to interpreting the methodological parameters underlying the evidence related to safety findings, this paper avoids unnecessarily complex or theoretical jargon and focuses solely on the relevant aspects. As this is not an epidemiology or statistics paper, the concepts are presented in plain English, without the use of equations or statistical terminology.

We advocate for the promotion of critical appraisal of evidence to facilitate the understanding of drug safety findings. To accomplish this, we outline the available data and methods to evaluate drug safety signals. This approach aims to provide PV professionals with sufficient knowledge to appreciate the fundamental concepts, employ appropriate terminology, engage in meaningful discussions with colleagues conducting safety data analyses, and critically evaluate evidence related to drug safety investigations. Some of these concepts might be applicable to controlled trials, observational data, or spontaneously reported individual case reports based on the context of the data collected.

## 2 Analytical insights in deciphering drug safety evidence (part 1: data integrity concepts)

The accurate interpretation of associations heavily relies on the validity of the results obtained from analyzing available data and the evidence derived from it. In order to evaluate if exposure to a medicinal product is associated with an adverse event, an interpretation of the available data and the methods used to analyze the data is necessary. However, threats to the validity of evidence can arise throughout the assessment process, highlighting the need for a heightened appreciation of data interpretation pitfalls. Potential sources of error include, among others, confounding, selection bias, information bias, and not investigating effect modification. It is also important to consider the role of data from different study arms and the uncertainties of the evidence in understanding the safety profile. Each of these factors has the potential to distort the interpretation of safety data and will be discussed in this Part 1 of the paper.

### 2.1 Confounding

**FIGURE 1:**
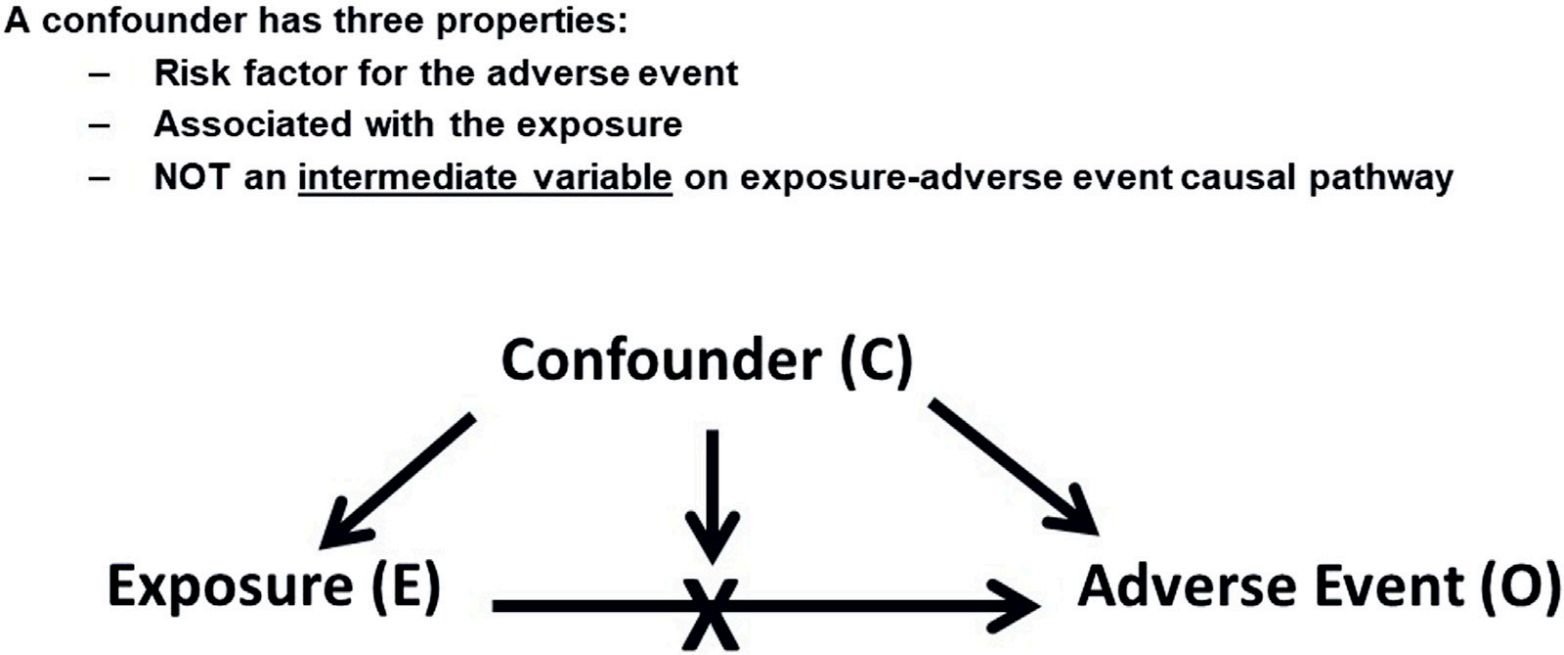
Confounding Factors. This diagram shows how a confounding factor (C) relates to both the exposure (E) and the adverse event (O), potentially leading to a spurious association between E and O.

Confounding occurs when an extraneous factor is associated with both the exposure to a drug and the outcome of interest (Rothman et al., 2008), potentially leading to a spurious association. Figure 1 illustrates this concept. Confounding, when left unaddressed, can have significant implications on the interpretation of drug safety findings. Its impact can be either overestimation or underestimation of the true association between exposure and an adverse event, potentially leading to the introduction of spurious associations or obscuring real ones. It is important to note that confounding does not necessarily require a concurrent presence of the confounding factor with the exposure to the drug of interest. For instance, a previous long-term exposure to a cytotoxic drug can potentially be a confounder for an AE that was reported if the AE caused by this drug appeared after starting a new one. This scenario might also be applicable when the treatment is switched between drugs resulting in potentially false attribution and AE to the latter treatment. Another potential caveat for switching scenarios will be explained later in this section under the “confounding by indication”.

With the increasing use of emerging data sources such as electronic medical records (EMR), social media, and patient-generated health data via wearables and mobile devices, it is important to appreciate the nuances of the data attributes and the inherent risk of bias and confounders associated with interpretation of data from these sources. For example, EMR data often includes more detailed patient information, which can improve confounder adjustment but also introduces complexities such as varying data quality and completeness as well as differences in coding practices. These variations must be considered when analyzing data from these sources.

A confounder possesses three key properties: It should be a risk factor for the adverse event (AE) under investigation, it should be associated with the drug exposure of interest, and importantly, a confounder should not function as an intermediate variable on the causal pathway between the exposure and the AE, as controlling for an intermediate variable would distort the actual effect that is being measured. For example, if acute myocardial infarction (MI) is being assessed as an adverse event, we should not consider the presence of hypertension in a patient’s medical history as a confounder if the drug being investigated is known to cause hypertension. Although hypertension is a risk factor for MI, it would be an intermediate variable in the causal chain in this particular scenario (Hammad et al., 2023). Effort should be made to quantify the extent of such association from the published literature to understand the likelihood of a particular risk factor exerting a confounding effect.

In general, some analytic techniques might help control for confounding effect in a study setting, e.g., in a PASS (Post-Authorization Safety Study), albeit it is more challenging sometimes with RWE (Real World Evidence) studies because of the lack of measurements of all pertinent confounders. The specifics of controlling for confounders would be provided by the colleagues analyzing the data. When adjusting for confounders through statistical models, it is important to examine the impact on the reported association, between the drug and the AE of interest, after accounting for each individual confounder. This step allows us to discern which confounder(s) may be primarily driving the observed results and assess the clinical plausibility. It should be noted that after adding each confounder, the association could also be unaffected. In other words, observe how the association between the drug and the AE changes after adding each confounder and if this change is reasonable in direction and magnitude.

Notably, the magnitude of the observed association between drug exposure and AEs is important as unmeasured confounding is particularly plausible to potentially explain small effects. The magnitude of risk may also influence regulatory decision making in classifying the risk as potential or identified, per the FDA NISS MAPP.^1^ Additionally, there is a legal dimension to the magnitude of the risk. Russellyn and Goldstein (2001),^2^ published a report where they reviewed 31 legal cases of toxicity to assess whether “*epidemiological data demonstrating a relative risk [RR] greater than 2 is required to meet the standard for proof (more likely than not) or to admit an expert’s opinion of causation*.” The report mentioned that the frequency of references to RR greater than 2 in judicial opinions appears to be increasing, although it is not yet widespread.

Another related concept is confounding by indication.^3^ Confounding by indication occurs when the indication or contraindication for treatment, as well as the severity of the disease, influence the decision to initiate, switch, or withhold certain treatments. This can confound the relationship between the treatment and a particular AE, making it challenging to isolate the true effect of the treatment itself. Proper consideration for confounding by indication is important to ensure accurate and unbiased assessments of treatment outcomes, the safety profile, and the benefit-risk. For example, a patient with risk factors for gastrointestinal bleeding has an increased probability of being prescribed COX-2 inhibitors. Such risk factors inherently predispose patients to subsequent bleeding. Consequently, a spurious association might emerge between the use of COX-2 inhibitors and gastrointestinal bleeding due to the common causes of both COX-2 prescription and subsequent bleeding, which are the gastrointestinal bleeding risk factors acting as confounders. Other examples include suicide or suicidal ideation secondary to depression, which may appear spuriously associated with SSRI treatment since the severity of depression influences the decision to initiate SSRI therapy. Similarly, acute myocardial infarction (AMI) secondary to advanced diabetes may seem spuriously linked to insulin use, as insulin is often prescribed at later stages of diabetes when the risk of AMI is already higher. Additionally, if renal failure is a known risk with some drugs in a particular class, this can lead to patients with relevant risk factors being channeled towards a specific drug within that class that is not associated with renal failure. In turn, this can create a spurious association between the alternative drug and renal failure due to confounding. In a study setting, appropriate study design and analysis should consider addressing these risk factors to account for their influence and mitigate the confounding effect (Yoshida et al., 2015).

It is worth noting that the confounding effect might also be encountered in some scenarios in randomized clinical trials (RCT) (Hammad et al., 2011). Conceptually, randomization used in clinical trials is primarily designed to mitigate the impact of pre-existing differences between study groups at the outset of a study by ensuring that these, measured and unmeasured, factors are evenly distributed across treatment groups and as such minimize potential for confounding. However, randomization alone cannot guarantee perfect balance between groups, especially in small sample sizes of high variable patient population where chance imbalances in important, measured and measured, confounding factors may occur. The tendency for achieving the balance between comparator groups increases as the sample size increases (Rothman et al., 2008). However, even if randomization initially successfully balances variables between groups, confounding might still play a role due to differential dropouts among other reasons. Because of this, we should not consider all RCTs equal in the weight of evidence. Critical assessment of the design and conduct of these trials is a prerequisite to appropriate decision making. More detailed information about the intricacies of various scenarios in drug safety can be found in Hammad et al., 2011 and CIOMS X report,^4^ but In short, confounding effect can arise due to the frailty of randomization caused by differential discontinuation between the comparison groups leading to an imbalance in the distributions of risk factors among study groups. Another contributing factor is the frailty of blinding, as the occurrence of some known drug-related AEs can unmask the assigned treatments, leading to increased awareness and surveillance for other AEs. Furthermore, subgroup analysis can introduce confounding because the comparability achieved by randomization in the main study groups may not extend to the subgroups (Groenwold, et al., 2009). Additionally, when pooling data from multiple RCTs without preserving randomization boundaries (i.e., without stratifying by study, also known as simple or “crude pooling”), summary estimates can be biased due to confounding by study (Hammad and Pinto, 2016). For example, in some scenarios, simple pooling of trial data can result in a phenomenon known as Simpson’s paradox leading to spurious results potentially reversing the association between a drug and an AE (Chuang-Stein and Beltangady, 2011; FDA guidance document, 2018^5^).

In addition to scenarios involving observational and controlled studies, the confounding concept might apply to the review of individual case safety reports (ICSRs) when investigating preliminary evidence for causality assessment and evaluating the role of pre-existing medical conditions and concomitant medications as potential confounders or alternate etiology for the AE. Being mindful of all these nuances will enable recognizing and appropriately accounting for confounding factors by PV professionals, enhancing the validity of their findings and providing more robust evidence for drug safety assessments.

### 2.2 Effect modification

**FIGURE 2:**
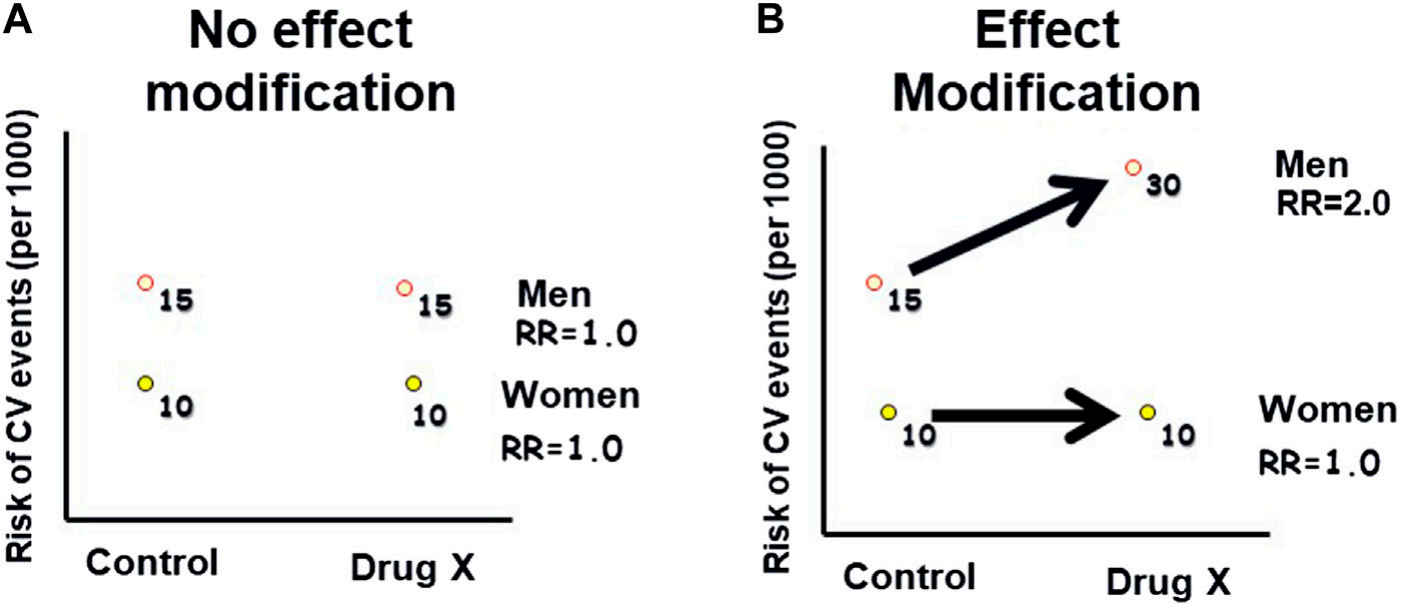
Hypothetical example of effect modification: This graph illustrates how the effect of an exposure (Drug X) on an outcome (Cardiovascular Events) varies across different levels of a third variable (gender), indicating effect modification. **(A)**: The risk of CV events is higher in men than in women, in both the Drug X and control groups. **(B)**: The risk of CV events is higher in men than in women, with a greater difference in the Drug X group compared to the control group. Therefore, gender modified the risk associated with the drug.

Effect modification, also referred to as “interaction” or “treatment effect heterogeneity,” occurs when the relationship between an exposure and an outcome differs across levels of a third variable, known as the effect modifier (Rothman et al., 2008). Figure 2 demonstrates this concept. Common effect modifiers include demographic factors such as gender or race, as well as variables like age, genetics, ethnicity, co-morbidity, and co-medication. Unlike confounding, effect modification is considered a clinically informative finding to patients and healthcare practitioners and should be reported for all levels of the effect modifier, rather than solely being adjusted for. In drug safety studies, effect modification can help identify subgroups of patients who may be particularly susceptible or resistant to certain AEs. It is important to request the assessment of effect modification on safety findings from the analytical study team, whether the study is controlled or observational. Analytically, assessing effect modification involves examining interaction terms in regression models or conducting subgroup analyses to understand how the drug’s safety profile varies across different subpopulations. Failing to evaluate effect modification can result in the observed safety effects representing the average effect across all levels of the effect modifier, masking potentially important variations in the safety findings. As an example of effect modification by race, some studies suggested reduced efficacy and increased cardiovascular and cerebrovascular morbidity and mortality with angiotensin-converting enzyme (ACE) inhibitors and angiotensin receptor blockers (ARBs) monotherapy in Black hypertensive patients (Helmer et al., 2018).

This phenomenon might also be encountered even in individual post-marketing reports when a synergistic effect on an AE is observed, indicating that the impact of the treatment on the AE is modified by other factors. Drug-drug interaction is an example of effect modification in drug safety. For example, moderate to strong CYP3A4 inducer, can reduce progestin or estrogen exposure to an extent that could decrease the effectiveness of hormonal contraceptives. Similarly, drugs that inhibit an enzymatic pathway of CYP may cause increased concentrations of other drugs metabolized by the same pathway, resulting in drug toxicity (McDonnell and Dang, 2013). Assessing and reporting effect modification is needed for a comprehensive understanding of the relationship between treatment and AEs, enabling tailored treatment approaches and improved patient care.

### 2.3 Selection and information bias

Various biases, including selection, recall, and information bias, can affect the validity of the findings of drug safety investigations. For instance, such biases might arise from flawed study designs or conduct. Many of these biases can be avoided or at least minimized through a well-principled design, e.g., in the case of RCTs. Methods like quantitative bias analysis (QBA) and sensitivity analyses can be utilized to evaluate the robustness of results and underlying assumptions (Petersen et al., 2021). Proper expertise is pertinent not only for conducting these studies but also for interpreting their results. Some of these biases might also come into play impacting the validity of the assessment of AEs in individual patients, not just in a study setting. In this section we will familiarize the readers with some of these sources of bias to take it into consideration when interpreting pertinent data.

Selection bias in drug safety can manifest during the inclusion process of patients and also throughout the follow up period of a study. In terms of inclusion, selection bias occurs when individuals self-select themselves for participation in a study, leading to a biased sample (Gajda et al., 2021) or when study design allows inclusion of subjects with specific conditions or risk factors. The “volunteer bias”^6^ arises when participation is associated with both the exposure and the outcome being investigated. For example, individuals who are more health-conscious, have advanced disease stages, or have experienced treatment failures may be more likely to volunteer for the study. As a result, the study sample may not accurately represent the general population, leading to biased results. Although, in a controlled clinical trial, randomization might neutralize the effect maintaining internal validity of the study, the external generalizability might not be as high. This issue can also occur in prospective observational studies or registries that depend on patients volunteering to be part of the activity. During the conduct of a study, selection bias can also occur, for example, if subjects are more likely to drop out from the placebo group compared to the drug group if they are not responding to the intervention. This phenomenon can result in informative censoring (Olivier and Prasad, 2024), where the reasons for dropout might be related to the outcome being studied. Consequently, the drug group may end up with an apparently higher proportion of patients with more severe conditions experiencing more AEs or *vice versa*, potentially biasing the observed treatment effect.

Information bias, also known as measurement bias or misclassification, can arise from inadequate or inaccurate measurement or classification of study variables such as exposures, outcomes, or confounders. This bias occurs when there are errors in capturing and categorizing the relevant information, leading to distorted or incomplete data ultimately affecting the validity of safety findings. Information bias can take two forms: non-differential and differential misclassification (Rothman et al., 2008).

Non-differential misclassification occurs when the degree of misclassification is similar across all study groups and is random in nature. This type of misclassification tends to bias the results towards the null, meaning it attenuates the observed association or fails to detect a safety finding, although some exceptions exist (Jurek et al., 2005). Non-differential misclassification is more likely to occur in drug safety studies compared to efficacy studies, as safety outcomes may be less precisely defined at the outset of the study than efficacy endpoints. For example, if a study investigating the association between a specific medication and the occurrence of liver toxicity, non-differential misclassification may occur if the diagnostic criteria for liver toxicity are inconsistently applied across the study population. If the misclassification is random and occurs to the same extent in both the exposed and unexposed groups, it would be considered non-differential misclassification. This could lead to an underestimation of the true association between the medication and liver toxicity, as the misclassification dilutes the observed effect measures. Additionally, when non-differential misclassification affects confounders, it can lead to residual confounding, meaning there is residual distortion in the association between the exposure and safety outcome even after adjusting for known confounding factors.

In contrast, differential misclassification occurs when the errors in measurement or classification of AEs differ between the comparator groups. This type of misclassification introduces a systematic bias that can distort the observed associations between a drug and an AE, making them appear stronger or weaker than they truly are, potentially leading to erroneous conclusions. For example, detection or surveillance bias is a type of differential misclassification (Hammad and Pinto, 2016). This bias can introduce spurious or overestimated associations in various scenarios. It arises when patients in one group have a higher probability of having the AE detected due to increased surveillance, screening, or testing of the outcome itself or an associated symptom. One example is when patients become inadvertently unblinded in clinical trials due to experiencing AEs known to be associated with the class of the studied intervention. For instance, in trials evaluating antidepressant drugs, patients may report a side effect of sexual dysfunction (Serretti and Chiesa, 2009), which can lead someone in the study staff to realize that the patient is in the active drug group, thus increasing the likelihood of close monitoring and capture of other AEs inflating the rate of overall AEs. Similar situations can arise with other medications, such as ACE inhibitors, where patients become unblinded because of reporting dry cough (Pinto et al., 2020), or with 5-alpha reductase inhibitors like finasteride, which may prompt patients to report gynecomastia (Hagberg et al., 2017).

Another instance of detection bias occurs when patients are subjected to targeted investigations or screening based on the known AEs associated with a particular drug. For example, in the case of Mirapex used for Parkinson’s disease and Restless Legs Syndrome, which has been linked to peripheral edema (Tan and Ondo, 2000), patients may receive more thorough monitoring for signs of congestive heart failure (CHF). This targeted surveillance can result in an inflated association between Mirapex and CHF. Similarly, drugs like entacapone (used for Parkinson’s) and rifampin (used to treat tuberculosis) have been associated with urine discoloration (Snider and Farer, 1977), leading to an increased likelihood of detecting prostate cancer due to enhanced screening efforts in the treatment group. Likewise, post-menopausal estrogen use may cause increased abnormal bleeding (De Medeiros et al., 2013), potentially inflating the observed association between estrogen therapy and endometrial cancer due to the additional investigation being done in the treatment group. Furthermore, the occurrence of many common AEs in the drug group might potentially lead to increased interactions between patients and healthcare practitioners. This heightened interaction may facilitate the detection and reporting of other AEs differentially in the drug group that may not have been captured otherwise, potentially leading to spurious associations.

Protopathic bias is another source of distortion of the drug-AE association (Hammad et al., 2008; Faillie, 2015). It represents a unique type of bias related to the temporal relationship between exposure and outcome. This bias can occur when the early symptoms of a disease prompt individuals to seek medical attention and subsequently receive exposure of interest, leading to spuriously assuming that the exposure caused the disease. For example, if a patient suffered from chest pain and was prescribed an NSAID, then the situation progressed to a myocardial infarction (MI), the NSAID might be inaccurately flagged as the cause of the MI. However, the chest pain was simply experienced during the prodromal stage of the MI.

Being aware of these sources of error in the evidence will help the PV professional investigate the potential scenario based on knowing the nuances of the indication and the drug being investigated, which will enable making informed decision when assessing safety signals.

### 2.4 Role of placebo groups in drug safety

The term “Placebo” was coined in the 18th century to describe the phenomenon of symptom improvement in patients who were administered inert substances with no anticipated therapeutic effect. The word “Placebo” originates from Latin and translates to “I shall please,” reflecting the concept of providing comfort to patients. In earlier times, placebos were administered when actual drugs were unavailable or when patients believed themselves to be sick, despite being healthy in reality (Miller et al., 2009). The placebo group serves as a comparison in controlled trials for assessing the safety profile of many drugs to determine drugs’ specific contribution to safety concerns. There was some ethical debate on the appropriateness of exposing patients to placebo in studies. Ellenberg and Temple (2000), of the FDA, concluded that placebo controls are ethical when delaying or omitting available treatment has no permanent adverse consequences for the patient and as long as patients are fully informed about their alternatives. The Declaration of Helsinki^7^ supports this notion in its Ethical Principles for Medical Research Involving Human Subjects.

One of the challenges facing trials with placebo groups is the “placebo response.” This refers to the phenomenon where patients experience improvements in their symptoms or outcomes after receiving a placebo, which is an inactive treatment with no anticipated therapeutic effect. The placebo response is an important consideration in clinical trials as it can impact the interpretation of treatment outcomes and the assessment of the true effects of the investigational drug. Understanding and accounting for the placebo response is essential for accurate evaluation of treatment effects and ensuring the validity of study results. Several factors contribute to the placebo response observed in medical interventions. Psychological and social characteristics of the individual patient also play a role, as patient beliefs, expectations, and personality traits can impact their response to treatment. Other factors might include misclassification or error in measurement, previous experiences with success or failure, the beneficial or harmful effects of associated standard medical care, informed consent processes, the nocebo effect (negative expectations leading to adverse effects), and regression to the mean (reversion of extreme values toward the average). Considering these multifaceted factors is relevant when assessing and interpreting the placebo response in clinical trials. Interestingly, studies have reported that certain placebos can elicit clinically relevant responses, which can have a notable impact on the perceived relative effectiveness of active treatments. This finding holds significance in the design and interpretation of clinical trials (Bannuru et al., 2015). Surprisingly, there are websites that sell placebos, further highlighting the unique nature of the publicity behind this concept.^8^

When there is a need to estimate background rates of AEs in a patient population, there may be a temptation to utilize data collected from placebo groups in individual RCTs or through meta-analysis of multiple trials, especially when epidemiological background data is not available. However, several caveats should be considered when using AE rates from placebo groups. Firstly, it is important to remember the factors that influence the placebo response, as this can impact the observed rates of AEs. Additionally, patients participating in clinical trials may not be representative of the general patient population or even those involved in other trials due to volunteer bias. Variations in the level of sickness of patients based on the phase of drug development, as well as differences in inclusion and exclusion criteria between trials, can further influence the estimation of background rates. Nuanced differences in trials design and conduct, such as the sampling domains used (especially cross many countries) or the presence of a “close monitoring” effect, can also impact observed AE rates. Differential reporting practices among trials, where some trials actively collect AEs in specific forms, may lead to higher rates in certain trials. The “standard of care” effect, which can vary between trials, and should also be considered. Additionally, placebo groups might not capture rare events adequately, particularly when the sample size is relatively small or the follow-up time is short. These caveats highlight the complexities and limitations associated with using AE rates from placebo groups to estimate background rates of AEs in a given patient population.

A relevant concept here is the “nocebo effect.” The name of this effect is derived from the Latin word meaning “to harm” and is the opposite of the placebo effect. It refers to a phenomenon where a negative outcome occurs due to the patient’s belief that the intervention will cause harm.^9^ In the context of drug safety, the nocebo effect is an important yet sometimes overlooked factor. AEs associated with the nocebo effect are often physically experienced by the patient and can be clinically diagnosable. Certain patient populations, such as those with anxiety, depression, or a pessimistic outlook, may be at a higher risk of experiencing nocebo effects.^10^

To mitigate the risk of the nocebo effect, the study staff should strike a balance in providing information about both the positive and negative effects of the treatment. Ensuring that the patient understands the rationale behind the treatment can also help reduce the likelihood of the nocebo effect. Careful consideration should be given to the framing of information during the informed consent process. Using a positive framing approach, such as highlighting that the majority of patients experience no difference between two drugs, while acknowledging that a small number may notice a difference, can be beneficial (Planès et al., 2016). In addition to patient education, it is essential to provide education to study principal investigators (PIs) as well. Enhancing their awareness and understanding of the nocebo effect can aid in effectively managing and minimizing its impact. By addressing the nocebo effect through a comprehensive approach that includes patient education, informed consent strategies, and education for study PIs, it is possible to mitigate the potential negative consequences associated with this phenomenon.

### 2.5 Uncertainty in evidence

Drug safety investigations often encounter uncertainties arising from factors such as sample size limitations, missing data, ambiguous AE definitions, and outcome measurement variability. These uncertainties can be categorized into four main groups: clinical, statistical, methodological, and operational (Forum on Drug Discovery, 2015). To mitigate some of these uncertainties and obtain more reliable estimates of drug safety, analytical methods like sensitivity analyses or imputation techniques might be employed in some situations, as applicable. Statistical uncertainty can sometimes be quantitatively assessed through hypotheses testing or confidence intervals in some scenarios aiming to quantify the magnitude of uncertainty based on a set of underlying assumptions that can be evaluated. The goal of addressing uncertainty is not to replace clinical judgment with an automatic process but to enhance and improve decision-making abilities. Identifying and addressing knowledge gaps effectively in the development program is a critical aspect of managing uncertainty. However, it is equally critical to understand and operate within the scientific boundaries of the available research tools and methods, ensuring that the information obtained is dependable and valid.

By definition, a safety signal represents an uncertain finding; it is the analysis of the signal that informs regulatory recommendations, not merely the presence of the signal. FDA regulators acknowledge that enhanced transparency in the early communication of safety signals inherently brings unavoidable uncertainty (Dal Pan and Temple, 2011). The primary challenge lies in promptly identifying these signals and evaluating them as efficiently as possible to minimize the period of uncertainty between the identification of a signal and its resolution (Dal Pan and Temple, 2011). In managing uncertainty, regulators commonly adhere to the “Precautionary Principle,” which dictates that actions should be taken to prevent or reduce morally unacceptable harm that is scientifically plausible yet uncertain (Eichler et al., 2013). However, the authors caution against excessive risk aversion or excessive demands for more data, as these may lead to diminished net health gains from drug research and development. To minimize this risk and provide a fair assessment of the interpretation of safety data, it is important to transparently acknowledge and address the sources of uncertainty during the evidence preparation and regulatory submission process.

Uncertainty also plays a role in benefit-risk (BR) assessment, where it is essential to consider several key factors. The BR decision-making process is complex encompassing both quantitative and qualitative dimensions. It is important to acknowledge that not all drugs are created equal, and the context in which they are used plays a vital role. Also, the dynamic nature of benefit-risk assessment underscores the importance of recognizing the clear disparity in the sources, timing, and nature of information regarding both benefit and risk. In the post-market phase, while information on risk continues to accumulate, data on benefits remains static, primarily driven by findings from trials conducted during development. Moreover, in addition to regulators and healthcare professional perspectives, a more appropriate way is needed to truly capture and incorporate the perspectives of patients in BR assessments. Understanding patient experiences, preferences, and priorities can provide valuable insights into the overall assessment process and help align it more closely with individual patient needs. The introduction of structured BR^11^ assessments (Hammad and Pinto, 2016) has necessitated explicit statements of uncertainties during the regulatory submission process. Regulatory guidance documents now provide comprehensive lists of considerations that can impact the level of uncertainty in product development^12^.

## 3 Analytical insights in deciphering drug safety evidence (part 2: data analysis concepts)

The assessment of the evidence in safety data relies on the appreciation of the scientific underpinnings of some of the evidence-based medicine (EBM) analytic tools as it relates to various sources of data. This encompasses various methodological and analytic concepts as well as some statistical techniques, which facilitates the correct interpretation of evidence and better understanding of drugs safety profiles. These concepts include measures of AEs frequency and related calculations, interpretation complexities of study results (e.g., study power, 95% confidence intervals and *p*-values, and role of meta-analysis), and performance metrics (e.g., sensitivity and specificity and positive and negative predictive values), which will be discussed in this Part 2 of the paper.

### 3.1 Measures of AEs frequency and related calculations

Assessing the frequency of adverse events (AEs) is essential in drug safety. This involves calculating AE rates, specifically incidence. Incidence measures the number of new cases of an AE that develop in a patient population during a specified time period, while prevalence represents the proportion of the population who have a specific AE during a specified time period, regardless of when it first developed. Because prevalence is determined by not only the number of patients affected but also their survival, it is a less useful measure in studying etiology compared to incidence (Ward, 2013).When investigating safety signals, it is important to focus on the incidence of AEs in the patient population at large rather than prevalence,^13^ as it reflects more accurately the anticipated background rate. The incidence can also be utilized to calculate the probability of encountering the observed number of AEs by chance among exposed patients, considering the background incidence and drug exposure. More specifics about this approach can be found in Hammad et al., 2023. Online resources^14^ ^15^ are available to facilitate such calculations efficiently.

An example of the utility of incidence during drug development is demonstrated by one of the requirements in the most recent FDA Safety Reporting Requirements for Investigational New Drugs (INDs)^16^. To ensure effective safety surveillance in the premarket phase, regulators encourage sponsors to focus reporting efforts on cases of serious and unexpected suspected adverse reactions (SUSARs) facilitating prompt risk mitigation in clinical trials during drug development. This FDA guidance document underscores the importance of considering the background rate of anticipated AEs in the reportability of certain types of SUSARs. The guidance particularly recommends aggregate analyses based on incidence for anticipated AEs when it is challenging to assess causality based on individual cases. This is especially important if the AEs are expected in the study population regardless of drug exposure, possibly due to the underlying condition under study or common background regimens. Additionally, such analyses are advised for AEs with higher incidences than stated in the protocol or investigator’s brochure. These analyses compare event rates in the study to historical incidence in the study population to identify potential imbalances. The guidance document acknowledges that eventually both expert clinical and statistical judgments are essential for interpreting these aggregate data and determining reasonable possibilities of causality based on available information.

The reporting rate (RepR) in the post-market phase is another measure of AE frequency. It is usually calculated by dividing the number of reported AEs associated with a specific drug by the estimated extent of exposure to the drug among the patient population within a specified time period. The numerator represents the count of reported AEs, often obtained from spontaneous reporting databases, while the denominator estimates the population exposure, often using sales data or prescription records. A general trend of increase in reporting of AE frequency during the first 2 years after introducing a new drug, followed by a subsequent decline, was observed and reported by Weber (1984). Analytically, the “Weber effect” might be considered when evaluating drug safety data in some situations to differentiate between true drug-related AEs and those influenced by reporting bias or increased surveillance. However, an FDA study did not detect the Weber effect despite observing shifts in the reporting rate over time (McAdams et al., 2008b). The authors investigated temporal trends in reporting for the total number of AE reports submitted to the FDA by consumers or healthcare providers. The study observed varying temporal patterns in reporting among the four drugs within the studied drug class. The number of reports was highest to varying degrees in the first year and subsequently declined. Another study examined the publicly available FAERS data for sixty-two drugs approved by the FDA between 2006 and 2010 and found no evidence of the Weber effect in the majority of the drugs (Hoffman et al., 2014).

Comparing RepR of AEs to background incidence rate (BIR) from independent data sources has limitations. Factors like healthcare provider awareness, patient reporting behaviors, and regulatory requirements, among others, can influence the reporting of AEs impacting the calculation of the RepR (García-Abeijon et al., 2023). Underreporting of AEs (McAdams et al., 2008a; García-Abeijon et al., 2023) and estimation of population exposure based on sales data can further impact the accuracy of the numerator and denominator, respectively. Therefore, the RepR might only serve as a rough proxy and should be interpreted cautiously. Hammad et al. (2023) proposed the calculation of observed-to-expected AE ratios during safety signal investigations, optimally comparing the observed AE RepR in a specific patient population to the BIR in the same population (e.g., if the AE was reported in patients taking a drug for diabetes then the incidence rate for comparison should be estimated in the diabetic population). This approach might be useful in identifying potentially drug related AEs when a higher RepR compared to the anticipated BIR may suggest a drug-AE association. Nonetheless, factors like media attention or litigation-driven reporting can stimulate reporting and lead to misleading results. Thus, it is important that any imbalance between the RepR and BIR should be thoroughly investigated taking into account these publicity factors (Hammad et al., 2023).

### 3.2 Interpretation complexities of study results in drug safety

The analysis and interpretation of study findings, whether derived from clinical trials or observational studies, play a key role in drug safety decision making. Methodological concepts from evidence-based medicine (EBM) such as study power, confidence intervals, and *p*-values serve to quantify the strength of evidence and evaluate the significance of observed associations between drugs and AEs. However, Ubbink et al. (2013) conducted a review of 31 surveys assessing the level of knowledge among healthcare professionals regarding these fundamental analytic concepts in EBM. Their findings revealed that a significant majority of physicians and nurses perceived their understanding of common analytic terms in EBM medicine to be inadequate. This highlights to unmet needs for these professionals.

Away from the mathematical intricacies underlying these concepts, it is important for PV professionals to be aware of key pointers on how to utilize EBM concepts in the context of drug safety assessment. For a deeper understanding and insights into the potential misinterpretation and misuse of some of these concepts, a reader-friendly publication by Greenland et al. (2016) provides comprehensive details and explanations.

#### 3.2.1 Study power

Study power reflects the ability of a study to detect an association if it truly exists.^17^ It is important to note that low power increases the risk of missing a safety signal, potentially overlooking important findings. However, it is worth noting that when studying thousands of exposed patients, statistically significant results may not always be clinically meaningful (Greenland et al., 2016). This is why medical judgment should always be utilized in interpreting any study findings.

Several factors influence the statistical power of a study. One such factor is the magnitude of the safety signal being investigated. A larger effect size or stronger association between the drug and the AE generally leads to increased study power for a fixed sample size. Another critical factor is the sample size. Larger sample sizes tend to enhance study power by providing more data points and reducing the impact of random variability. Additionally, the background rate of AE in the patient population can affect study power. If the background rate of AE is higher, it can potentially increase the power of the study to detect any additional AEs associated with the drug exposure.

It is worth noting that most studies designed for efficacy are not powered for safety endpoints unless such a study has safety as the primary objective. Therefore, it is important not to dismiss the presence of a safety concern solely based on the absence of observed AEs in a particular study. There can be various reasons for the apparent absence, which need to be carefully considered. Factors such as the study population’s characteristics, including the specific inclusion and exclusion criteria, can influence the detection of AEs. Additionally, the length of follow-up might not be sufficient to capture all potential events, and the study’s power could be limited due to factors such as a rare event, low background event rate, small sample size, or small magnitude of risk. Some investigators are tempted to calculate *post hoc* power for studies. These estimates are sometimes requested in an attempt to promote more rigorous designs. However, they should not be done because it was reported that they have been shown to be logically invalid and misleading (Dziak et al., 2020). Considering and understanding these factors is imperative when designing studies to ensure optimal power and accurate interpretation of safety findings.

#### 3.2.2 95% confidence interval and *p*-value

95% confidence intervals (CI) play an essential role in the interpretation of the clinical research data in drug development as they provide a range of plausible values within which the true population parameter lies, such as the risk of a particular AE.^18^ This is based on the understanding that we are sampling a patient population with inherent variability to estimate the “truth” about the drug-AE association under investigation. The width of the CI reflects the level of uncertainty, with wider intervals indicating greater uncertainty for a fixed error. The uncertainty arises from the variability among the sample members within the patient population. The width of the CI is influenced, in addition to the sample size, by other factors such as the magnitude of risk and the data noise, encompassing all sources of variation.

It is important to note that the CI is not a probability in itself, but in repeated studies with identical sampling, 95% of the CIs will include the true population parameter. In simpler terms, if 100 identical samples of patients were taken and, say, their mean blood pressure was measured, approximately 95 of those CIs would contain the true mean blood pressure of the sampled population. The 95% CI reflects both the effect size of the parameter being measured (represented by the point estimate) and the precision (represented by the width of the interval), which tends to be wider for smaller sample sizes with higher variance. The *p*-value, on the other hand, is confounded as it reflects both the effect size and precision combined. In general, CIs are considered more informative than hypothesis tests and *p*-values as they shift the focus from the null hypothesis to the full range of effect sizes compatible with the data (Greenland et al., 2016). A *p*-value is the measure of probability that the null hypothesis was rejected when in fact the null hypothesis is true.^19^ The smaller the *p*-value, the lower the statistical compatibility with the null (Wasserstein and Lazar, 2016). However, caution should be exercised in relying on the *p*-value. The widespread use of “statistical significance” (generally interpreted as “*p* ≤ 0.05”) as a license for making a claim of a scientific finding (or implied truth) leads to considerable distortion of the scientific process (Wasserstein and Lazar, 2016).

When assessing the results of two studies or groups, if the 95% CI of one study contains the point estimate from the other group or study, the *p*-value for the difference between the results of the two studies will not reach significance (Greenland et al., 2016). For instance, a study reported a sixfold elevation in the risk of persistent pulmonary hypertension in newborns (PPHN) among mothers exposed to antidepressant drugs during pregnancy compared to unexposed mothers (Chambers et al., 2006). However, a subsequent study reported about two and half fold elevation in the risk (Källén and Olausson, 2008). These differences in risk estimates (6.1; 95% CI, 2.2–16.8 vs. 2.4, 95% CI 1.2–4.3, respectively) might raise doubts among some scientists regarding the safety signal altogether. However, understanding that the risk estimate of the second study falls within the 95% CI of the first study demonstrates that, although some differences in risk estimates can be attributed to study design factors, the overall results of the two studies might be considered “consistent” and reflect the increased risk of PPHN.

A relevant concept here is multiplicity, which is important for PV professionals to know when it comes to safety as we calculate many potential associations between drug exposure and adverse events in a particular study. Therefore, the findings are susceptible to type I error inflation (in simpler terms, it means that there is a higher chance of wrongly saying something is true when it is actually not) and making incorrect conclusions about study findings. Although in practice, no adjustments are made for multiplicity for most trials due to the inherent exploratory nature of the safety findings in these trials as well as the application of the “Precautionary Principal” following by many regulators (Eichler et al., 2013), it is important to understand the concept of multiplicity. This concept highlights the risk of inflating the likelihood of incorrect conclusions, underscoring the importance of considering the totality of evidence rather than focusing solely on statistical significance.

#### 3.2.3 Rule of 3

This rule is an important concept for PV professionals to be familiar with, particularly in scenarios where a specific AE of interest is not observed during drug development trials. As discussed in the study power section, the absence of a particular AE does not imply that the drug is entirely free of AEs. To establish an upper limit on the risk of this AE, the rule of 3 is employed, per regulatory requirements. The rule suggests that if no events are found, the upper limit of the 95% confidence interval (CI) for the risk of the AE can be estimated at 3 divided by the sample size (3/n) of the patient population that were studied. Although the origin of this rule remains unknown, it was first discussed in medical literature in 1975 (Rumke, 1975; Hanley and Lippman-Hand, 1983). It is applicable to subgroups as well, e.g., to calculate the upper limit of the rates of AEs in age and gender subgroups.

The rule of 3 also serves a purpose in assigning frequencies to certain AEs that are only reported in post-marketing surveillance. While some PV professionals may consider using exposure data to calculate the frequency, regulatory guidance emphasizes the use of the rule of 3.^20^ The guidance explicitly advises against using reporting rates from a spontaneous reporting system to assign frequency categories. However, a potential challenge arises as this approach may lead to paradoxical results, suggesting higher frequencies for some unobserved AEs than they actually are (Crowe et al., 2013).

It is important to recognize a limitation of the rule when the background rate of the AE is significantly lower than the upper limit of risk that can be ruled out. For example, say liver failure is suspected in drug with a development program involving 6,000 patients followed for 6 months (equivalent to 3,000 person-years). Applying the rule of 3 would yield an upper 95% CI for liver failure of 1/1,000 person-years. However, if the background rate of liver failure is 1/100,000 person-years, the data can only rule out a rate that is 100 times higher than the background rate. This underscores the limitations of the rule in certain scenarios. It should be noted that using Bayesian probabilities is becoming increasingly popular where the probability of observing a number of events based on the historical data is calculated, although it has its own set of limitations (Van de Schoot et al., 2014).

#### 3.2.4 Meta-analysis

Meta-analysis is another analytic concept that PV professionals should be familiar with. It involves systematically reviewing and synthesizing data from multiple RCTs to assess treatment effects comprehensively. In pharmacovigilance, pooling data from RCTs might be valuable as many AEs are rare and there is a need to characterize the effect of drugs from a broader and larger population – often times done on a class of drugs (Hammad et al., 2006). Meta-analyses that investigate safety concerns have gained increasing attention and influence in clinical and regulatory decision-making. Examples include meta-analyses examining cardiovascular AEs associated with rosiglitazone (Nissen and Wolski, 2007) and tiotropium (Singh et al., 2008), mortality rates associated with cefepime (Yahav et al., 2007), and suicidality associated with antidepressants (Hammad et al., 2006A; Hammad et al., 2006B, Stone et al., 2009; Pamer et al., 2010).

An important aspect in the assessment process of a meta-analysis is to confirm the complete capture of all trials investigating the safety signal of interest. Systematic reviews serve as a cornerstone in evidence-based medicine, providing a comprehensive synthesis of existing evidence on a particular topic. By systematically searching, selecting, and appraising relevant studies, systematic reviews aim to minimize bias, increase transparency in evidence synthesis, and improving the validity and generalizability of the meta-analytic findings. In this regard, one of the challenges to put in mind is that some published trials might be “silent” on reporting the safety profile of the studied drug, but it does not mean that no AE were observed during the trial (Ioannidis and Lau, 2001).

Conceptually, meta-analysis is most dependable when trials share similar designs and patient populations, with uncertainty in effect size often arising from inadequate statistical power. PV professionals must meticulously assess differences in trial design, patient demographics, and the presence of heterogeneity to draw meaningful conclusions from meta-analyses regarding drug safety. It is crucial to scrutinize the quality and the nuances of individual trials’ conduct to identify potential threats to validity. This is because some trials included in meta-analyses evaluating safety issues may be susceptible to biases inherent in retrospective observational studies, as discussed in the “confounding” section of this paper (Hammad et al., 2011). Pertinent medical knowledge and judgment of the PV professionals are paramount for success in this assessment. The CIOMS X guidance offers detailed recommendations on evidence synthesis and meta-analysis in the context of drug safety.^21^ Additionally, guidance to the assessment can be found in Hammad et al., 2011, where the authors outline considerations relevant to the evaluation of drug safety findings derived from meta-analyses of RCTs. In short, the first set of considerations focuses on the design and conduct of individual RCTs, highlighting issues such as the frailty of randomization and blinding, challenges in ascertaining AEs due to varying definitions and documentation practices, and difficulties in accurately assessing drug exposure. The second set of considerations pertains to the design and conduct of meta-analyses, addressing issues like publication bias, trial selection biases, challenges in integrating trials with differing designs or protocols, and statistical concerns such as type-I error inflation and subgroup analyses. These methodological nuances can impact the reliability and interpretation of drug safety findings, potentially leading to over- or underestimation of drug risks.

Hammad et al., 2013, examined a selection of published meta-analyses, particularly those concerning drug safety, which were published in prestigious scientific journals. A preliminary reporting framework was devised, integrating the Preferred Reporting Items for Systematic reviews and Meta-Analyses (PRISMA) guidelines with additional elements essential for evaluating drug safety meta-analyses. Most of the published meta-analyses reviewed (60%) failed to cover the majority (80%) of additional reporting elements necessary for assessing the validity of drug safety findings. Some of these elements were not addressed in any of the meta-analyses included in the review (Hammad et al., 2013). Although the PRISMA statement (Preferred Reporting Items for Systematic Reviews and Meta-Analyses) offers guidelines for meta-analysis authors to ensure comprehensive and transparent reporting, its emphasis primarily lies on efficacy rather than safety. Consequently, to address this gap, a PRISMA harms checklist has been developed. Its purpose is to enhance the reporting of harms in meta-analyses, thereby fostering a more comprehensive and equitable assessment of the benefits and risks associated with health interventions (Zorzela et al., 2016).^22^

Additionally, developing a solid understanding of the basic concepts of meta-analysis analytics is an essential skill for PV professionals to effectively evaluate the evidence and interpret the results. Meta-analytic techniques to analyze the pooled data across trials have implication on the interpretation. For example, it is important to appreciate that we are asking different questions when using the fixed effect model *versus* the random effects model in meta-analysis. The fixed effect model aims to determine the best estimate of the treatment safety effect, while the random effects model focuses on identifying the average treatment effect. The weight of trials is an integral consideration when comparing fixed effect and random effects approaches in meta-analysis. In the fixed effect approach, the assumption is that the true safety effect being estimated remains constant across trials. The observed variation between trials is attributed to sampling error, implying that if all trials were sufficiently large, they would yield the same treatment effect. To minimize the variance around the true effect within each trial, weights are assigned. Larger trials are given higher influence in the analysis, as they are presumed to provide more precise estimates due to their larger sample sizes (Dettori et al., 2022).

In contrast, the random effects approach assumes that the true safety effect may vary around an average within a distribution and that there is heterogeneity among trials. Unlike the fixed effect approach, the random effects model takes into account two sources of variation or “uncertainty”: within trials and between trials. Consequently, the weights assigned in the random effects model are more balanced between trials, regardless of their size. In other words, larger trials do not exert as much influence as in the fixed effect model. This approach is particularly suitable when heterogeneity is present or suspected. Due to the incorporation of two sources of variation, the 95% confidence interval is wider, i.e., with a lower likelihood of statistical significance. The risk estimate obtained in the random effects model can be either larger or smaller than that in the fixed effect model, depending on the extent and direction of the heterogeneity (Dettori et al., 2022).

To provide comprehensive results, the PV professional should recommend getting the findings from both models for comparison to assess if they differ significantly. As for determining which model to believe, it depends on the specific research context, clinical judgement, and the underlying assumptions of each model, and hence the significance of the explanation provided here about the underlying premise of each approach.

Another aspect to consider is how the meta-analysis study reports the risk estimate of a particular AE. Caution must be exercised when interpreting results of meta-analyses of drug safety, especially when considering various metrics of association such as risk differences (RD), hazard ratios (HRs), odds ratios (ORs), relative risks (RRs), and others. Each of these metrics offers different insights into the relationship between drug exposure and AEs, and their interpretation requires careful consideration of the study design, population characteristics, potential biases, and methodological limitations. For example, while RDs offers a straightforward measure of absolute risk reduction or increase, HRs, RRs and ORs are relative measures that may be influenced by the timing of events and the duration of follow-up and like other metrics, they may be susceptible to confounding factors if not properly adjusted for covariates. Therefore, when synthesizing evidence from multiple studies in a meta-analysis, it is important to assess the consistency of results across different metrics of association and consider the strengths and limitations of each approach in order to derive meaningful conclusions about the studied AEs.

It should be noted that caution is warranted when assessing attempts to conduct meta-analyses of observational studies in pharmacovigilance. In the field of pharmacoepidemiology, numerous observational studies may link certain drugs to AEs. Generally, when multiple studies examine the same drug-adverse reaction combination, there may be a temptation to conduct a meta-analysis to obtain a more “conclusive” answer. However, the belief that disparate study results can be combined to reveal the true or a more precise effect size through meta-analysis holds true only under specific conditions. As mentioned earlier, meta-analysis is most valid when studies share similar designs and patient populations. Yet, this assumption often does not hold true for observational studies. Statistical power is rarely the issue, and study results are typically heterogeneous due to numerous factors (other than genuine differences between subpopulations of patients), including differences in methods and adjustment for confounding. Applying meta-analysis to observational studies does not necessarily yield a more accurate estimate of effect size but rather might provide an “average” of residual confounding among studies. Additionally, given the vast array of indications for the studied drugs’ classes, it is difficult to decipher the effect of confounding by indication on a particular AE as reported in an observational study. Moreover, publication bias and confirmation bias can skew results, with negative outcomes frequently underreported. Tests for publication bias may not always detect it, and correcting for it, if present, could significantly alter results, suggesting a smaller effect size than the one reported.

### 3.3 Performance metrics (sensitivity and specificity, positive and negative predictive values)

Performance metrics such as sensitivity, specificity, positive predictive value (PPV), and negative predictive value (NPV) play a significant role in drug safety. They are relevant to the validation effort underlying different aspects in working up a safety signal: confirming alignment of an AE with a clinical case definition and assessing evidence for a causal association with the drug (Hammad et al., 2023).

First, let us review some of the definition and the trade-offs between these metrics. In the context of AEs, sensitivity measures the ability of a case definition or diagnostic test to accurately identify true positive cases, while specificity measures the ability to accurately identify true negative non-cases. Positive and negative predictive values estimate the probability that a positive or negative finding accurately predicts the presence or absence of the clinical condition of interest, respectively. Sensitivity and specificity are theoretical concepts that rely on knowing the true disease status, while PPV and NPV have direct clinical relevance^23^ as they are associated with encountering false positive and false negative cases (Monaghan et al., 2021). Understanding the trade-offs among these metrics is essential. Higher specificity is linked to higher PPV, indicating lower false positives, while higher sensitivity is associated with higher NPV, suggesting lower false negatives. The prevalence of a disease in the target population impacts PPV and NPV, with higher prevalence increasing PPV and decreasing NPV. Additionally, PPV is more influenced by specificity, and lower specificity has a greater impact on misclassification when clinical outcomes are uncommon. These facts are relevant to drug safety since the rates of most AEs are low. Therefore, using more specific definitions and tools is necessary for detecting true safety findings.

Examples of clinical conditions with validated diagnostic criteria, which aids in identifying true cases, include RegiSCAR scoring for DRESS with PPV of 87% (Kardaun et al., 2007; Kardaun et al., 2013; Kardaun et al., 2014), ADAMTS13 activity assay for Thrombotic Thrombocytopenic Purpura (TTP) with PPV of 91% (Barrows and Teruya, 2014), and Yamaguchi criteria for Adult-Onset Still’s Disease (AOSD) with PPV of 87.7 (Yamaguchi et al., 1992; Fautrel et al., 2002). An example of a validated causality assessment tool is the RUCAM (Roussel Uclaf Causality Assessment Method), which was validated to assess causality of cases of Drug Induced Liver Injury in clinical trials, where more information about liver enzymes is expected to be available. It had PPV of 93% and NPV of 78% (Bénichou et al., 1993; Danan and Bénichou, 1993). On the other hand, PV-RUCAM, which was validated for assessing post marketing DILI cases, had PPV = 25% and NPV = 100% (Scalfaro et al., 2017). So, the PV professional should always review the original papers that developed and validated the criteria to understand the clinical context for its development, how to apply it, and the implications on safety data interpretation. For example, knowing the PV-RUCAM performance metrics means that when assessing the DILI causality in a particular patient population, the false positive rate is going to be high, 75% (low PPV). Conversely, cases that do not fulfill the criteria are all true negative (high NPV). Therefore, while the PV-RUCAM is particularly good at ruling out the DILI causality (as indicated by the high NPV), it is less dependable at confirming its presence (as indicated by the low PPV).

These metrics are also pertinent to MedDRA search criteria. Using broad MedDRA codes (e.g., broad SMQs) increases sensitivity but reduces specificity, making it suitable for casting a wider net to capture more potential cases during initial assessment. However, misclassification becomes a challenge in comparative assessments as it might lead to attenuating safety signals when analyzing clinical trial data, using external databases for quantitative signal of disproportionate reporting, or comparing rates across different PBRER (Periodic Benefit-Risk Evaluation Report) intervals. Conversely, using focused Preferred Terms (PTs), as a second step in the search, increases specificity, reducing false positives and making it suitable for identifying and defining the AE of interest in a safety signal. Striking a balance between sensitivity and specificity depends on the objective of the search in the safety database.

Another utility of performance metrics is assessing the feasibility of a screening requirement in a study, such as to exclude vulnerable patients to certain AEs. Screening test performance should align with the purpose of using it, considering key metrics such as sensitivity, specificity, and PPV (Dobrow et al., 2018). The metrics provide a way to assess feasibility of identifying vulnerable patients based on the prevalence of the screened condition and the PPV of the screening tool itself. If the background rate of, say, suicidal ideation/behavior, in the patient population is very low, even a screening test with very high sensitivity and specificity will have a low positive predictive value if applied to all patients, while having the potential to significantly add to patient burden. An alternative is to target symptomatic patients to increase the PPV of vulnerable patients identification, which improves screening efficiency.

In conclusion, performance metrics are essential for evaluating the effectiveness of clinical definitions in drug safety as well as causality assessment. Sensitivity, specificity, PPV, and NPV play key roles in validating diagnostic tools, evaluating safety signals, setting MedDRA search criteria, and assessing the feasibility of screening requirements. Understanding these metrics and their trade-offs enhances the accuracy and reliability of drug safety assessments, ultimately leading to improved patient care and outcomes.

## 4 Commentary

In this paper, we delve into the scientific underpinnings of critical analytical considerations for interpreting drug safety evidence gleaned from clinical trials, observational data, and spontaneously reported individual case reports. Our aim is to shed light on their appropriate application and inherent limitations, empowering drug safety and healthcare professionals with practical insights to effectively interpret evidence around AE. This endeavor is poised to enhance the accuracy and reliability of drug safety assessments, thereby fostering evidence-based decision-making in healthcare.

Understanding analytical considerations in the interpretation of scientific evidence in drug safety might be tied to the concepts of repeatability, replicability, and reproducibility. Repeatability in drug safety refers to the ability to consistently reach the same conclusions when the same team conducts evidence assessments using the same approach and type of data repeatedly. This underscores the importance of methodological consistency within assessment teams to minimize variability and enhance the reliability of safety findings. Replicability, conversely, pertains to reaching similar conclusions when different teams of safety professionals assess the same evidence, emphasizing the necessity of appreciating the scientific boundaries of methodology interpretation across teams. Reproducibility extends this concept further by assessing whether different teams, across diverse scientific settings, can reach similar conclusions, highlighting the need for comprehensive methodological understanding to ensure the generalizability and validity of evidence interpretation.

Artificial Intelligence (AI) offers promising advancements in drug safety, such as standardizing signal detection and automating routine tasks (Hammad et al., 2023). However, AI also introduces challenges. AI internal algorithms can be viewed as a “black box” making it difficult to understand how decisions of the AI models are made, which can lead to misguided conclusions if the models are not properly validated. Moreover, biases in training data can result in biased outputs. Therefore, while AI has the potential to enhance drug safety procedures, it must be applied cautiously at this stage, with a clear understanding of its limitations and continuous validation against robust, well established methodologies.

Collaboration of PV professionals with pertinent experts, such as epidemiologists and statisticians, is crucial in interpreting safety data, given its integral role in benefit-risk assessment. As novel data sources and methodologies emerge, it is essential to stay updated and adapt analytic approaches accordingly. Future research should explore emerging novel approaches, such as artificial intelligence, and incorporate advances in statistical techniques and data science. Interdisciplinary research efforts that bring together experts from various fields can significantly contribute to the evolution of analytic approaches in drug safety research.

## Data Availability

The original contributions presented in the study are included in the article/Supplementary Material, further inquiries can be directed to the corresponding authors.

## Footnotes

1
https://www.fda.gov/media/137475/download

2
http://www.jstor.org/stable/29762698

3
https://catalogofbias.org/biases/confounding-by-indication/

4
https://cioms.ch/working_groups/working-group-x/

5
https://www.fda.gov/regulatory-information/search-fda-guidance-documents/meta-analyses-randomized-controlled-clinical-trials-evaluate-safety-human-drugs-or-biological

6
https://catalogofbias.org/biases/volunteer-bias/

7
https://www.wma.net/wp-content/uploads/2016/11/DoH-Oct2008.pdf

8
https://placebo.com.au/

9
https://www.cancer.gov/publications/dictionaries/cancer-terms/def/nocebo-effect

10
https://medsafe.govt.nz/profs/PUArticles/March2019/The%20nocebo%20effect.htm

11
https://www.fda.gov/media/115672/download

12
https://www.fda.gov/media/152544/download

13
https://www.nimh.nih.gov/health/statistics/what-is-prevalence#:∼:text=Incidence%20is%20a%20measure%20of,they%20first%20developed%20the%20characteristic

14
https://stattrek.com/online-calculator/binomial.aspx

15
https://stattrek.com/online-calculator/poisson.aspx

16
https://www.fda.gov/regulatory-information/search-fda-guidance-documents/safety-reporting-requirements-inds-investigational-new-drug-applications-and-babe

17
https://www.scribbr.com/statistics/statistical-power/

18
https://www.nlm.nih.gov/oet/ed/stats/02-950.html

19
https://www.nlm.nih.gov/oet/ed/stats/02-940.html

20
https://health.ec.europa.eu/system/files/2016-11/smpc_guideline_rev2_en_0.pdf

21
https://cioms.ch/working_groups/working-group-x/

22
https://www.equator-network.org/reporting-guidelines/prisma-harms/

23
https://www.acm.org/publications/policies/artifact-review-badging
